# The usefulness of the genetic panel in the classification and refinement of diagnostic accuracy of Mexican patients with Marfan syndrome and other connective tissue disorders

**DOI:** 10.17305/bb.2023.9578

**Published:** 2024-04-01

**Authors:** Giovanny Fuentevilla-Álvarez, María Elena Soto, Yazmín Estela Torres-Paz, Sergio Enrique Meza-Toledo, Gilberto Vargas-Alarcón, Nadia González-Moyotl, Israel Pérez-Torres, Linaloe Manzano-Pech, Ana Maria Mejia, Claudia Huesca-Gómez, Ricardo Gamboa

**Affiliations:** 1 Department of Physiology, Instituto Nacional de Cardiología Ignacio Chávez, México City, Mexico; 2 Department of Biochemistry, Escuela Nacional de Ciencias Biológicas, Instituto Politécnico Nacional (IPN), México City, Mexico; 3 Department of Immunology, Instituto Nacional de Cardiología Ignacio Chávez, México City, Mexico; 4 Cardiovascular Line in American British Cowdray (ABC) Medical Center, México City, Mexico; 5 Research Direction, Instituto Nacional de Cardiología Ignacio Chávez, México City, Mexico; 6 Department of Cardiovascular Biomedicine, Instituto Nacional de Cardiología Ignacio Chávez, México City, Mexico; 7 Department of Blood Bank, Instituto Nacional de Cardiología Ignacio Chávez, México City, Mexico

**Keywords:** Marfan syndrome (MFS), other connective tissue disease, next-generation sequencing (NGS), genetic mutations, cardiovascular damage, Mexican patients

## Abstract

Marfan syndrome (MFS) is a multisystem genetic disorder with over 3000 mutations described in the fibrillin 1 (*FBN1)* gene. Like MFS, other connective tissue disorders also require a deeper understanding of the phenotype–genotype relationship due to the complexity of the clinical presentation, where diagnostic criteria often overlap. Our objective was to identify mutations in patients with connective tissue disorders using a genetic multipanel and to analyze the genotype–phenotype associations in a cohort of Mexican patients. We recruited 136 patients with MFS and related syndromes from the National Institute of Cardiology. Mutations were identified using next-generation sequencing. To examine the correlation between mutation severity and severe cardiovascular conditions, we focused on patients who had undergone Bentall–de Bono surgery or aortic valve repair. The genetic data obtained allowed us to reclassify the initial clinical diagnosis across various types of connective tissue disorders. The transforming growth factor beta receptor 2 (*TGFBR2)* rs79375991 mutation was found in 10 out of 16 (63%) Loeys–Dietz patients. We observed a high prevalence (65%) of more severe mutations, such as frameshift indels and stop codons, among patients requiring invasive treatments like aortic valve-sparing surgery, Bentall and de Bono procedures, or aortic valve replacement due to severe cardiovascular injury. Although our study did not achieve precise phenotype–genotype correlations, it underscores the importance of a multigenetic panel evaluation. This could pave the way for a more comprehensive diagnostic approach and inform medical and surgical treatment decision making.

## Introduction

Marfan syndrome (MFS) is one of the most common inherited disorders affecting connective tissue, with primary clinical manifestations in the skeletal, ocular, and cardiac systems [1]. Reduced survival is primarily due to aortic complications, such as aortic root dilatation and dissection. Other cardiovascular issues like aortic dissection, mitral valve prolapse, aortic rupture, tricuspid valve prolapse, and proximal pulmonary artery enlargement [2] are the most significant causes of morbidity and mortality in this pathology and occur in up to 25% of patients during childhood. Mutations in the fibrillin 1 (*FBN1*) gene, which encodes fibrillin-1, are the leading cause of MFS and can be identified in 91%–95% of subjects with classic Marfan [3]. MFS is a multisystem genetic disease characterized by autosomal dominant inheritance and high penetrance, which can be considered 100% [1]. Patients with *FBN1* mutations display a range of phenotypes from mild to severe. Over 3000 mutations in the *FBN1* gene have been described in patients with MFS [4]. However, the relationship between phenotype and genotype still requires clarification due to the complex clinical presentation of MFS and the significant overlap of diagnostic criteria with other Marfan-like disorders. Mutations in a wide variety of genes, such as *TGFBR1*, transforming growth factor beta receptor 2 (*TGFBR2*), *SMADs*, *SMARD*, *COL4A3*, *COL4A2*, *COL5A1*, *FBN2*, *ACTA2*, *MYH11*, and SKI, are associated with other Marfan-like syndromes [5], including Loeys–Dietz (LDS), Beals–Hecht (BHS), Ehlers–Danlos (EDS), Weill–Marchesani (WMS), Shprintzen–Goldberg (SGS), Stickler syndrome (StS), and Mitral Valve, Myopia, Skin, and Skeletal disorder (MASS) [6]. Classifying patients into the various MFS variants is challenging for two reasons. Firstly, the clinical criteria are specific to MFS and not to associated syndromes [7]. Secondly, mutations causing Marfan-like syndromes affect the same signaling pathways [8], resulting in patients with numerous overlapping physical and clinical characteristics. The last revision of the Ghent nosological criteria occurred in 2010. Expert consensus concluded that a patient meeting more than two criteria can be classified as having MFS, while other connective tissue disorders can be ruled out [9].

The course and progression of cardiovascular damage in patients with MFS and other MFS-like syndromes vary. For those experiencing aortic dilation or dissection, surgical intervention is often unavoidable and sometimes urgent [10]. In cases of aortic dissection, substitutive surgical techniques are generally the preferred option. However, for uncomplicated individuals, the decision to proceed with elective surgery is based on the annual rate of aortic diameter increase and a maximum diameter between 4 and 4.5 mm. The 2022 clinical guidelines recently emphasized the importance of timely diagnosis through genetic tests, particularly using next-generation sequencing (NGS) technology. Such tests allow for personalized follow-up, timely treatment, and assist surgeons in selecting the appropriate surgical technique [11, 12]. Surgeons carefully evaluate aortic aneurysms across all anatomical segments to choose the suitable surgical procedure. The treatment decision hinges on the risk of aneurysm rupture and the patient’s life expectancy. In cases where patients show symptoms of an expanding aneurysm alongside aortic dilation, urgent surgery is generally indicated. Interdisciplinary heart teams consider all relevant surgical guidelines in such cases [13, 14]. On the other hand, data obtained through NGS can identify mutations in each gene associated with connective tissue disorders. It also checks for mutations in other genes that may contribute to cardiomyopathies, arrhythmias, and valvular and structural heart diseases. A patient with MFS may have a mutation in the *FBN1* gene but could also have coexisting mutations in genes coding for other proteins. This could explain the phenotypic diversity and heterogeneity in cardiovascular complications observed in these conditions.

Therefore, this study aimed to evaluate mutations in a panel of 174 genes associated with aortic damage, cardiomyopathies, arrhythmias, and structural heart diseases. The goal was to compare the data obtained with clinical diagnoses and to improve genotype–phenotype correlations in a cohort of Mexican patients.

## Materials and methods

### Research population

In a prospective observational study, we enrolled 136 Mexican patients affected by a broad spectrum of connective tissue diseases with cardiovascular manifestations. An expert rheumatologist initially evaluated these patients and classified them according to the 2010 Ghent criteria, requiring more than two criteria for classification. These criteria include: A positive family history for MFS (FH), aortic dilatation (AoD), ectopia lentis (EL), a systemic score (SS) greater than 7/20, and five positive demonstrations of a causative mutation in the *FBN1* gene. Meeting any two of these criteria strongly suggests the presence of the syndrome. Various imaging studies were conducted, starting with an initial echocardiographic evaluation by a cardiologist to assess aortic dilation or dissection, as well as mitral and aortic valve prolapse and tricuspid valve prolapse in the apical four-chamber plane. All patients underwent comprehensive imaging evaluations of cardiovascular damage through either magnetic resonance imaging or computed tomography. For the purpose of this study, Mexican patients were defined as individuals with at least three generations born in Mexico.

### Patients who required surgical procedure

Patients were scheduled for surgery if they had an aortic dissection or dilation measuring ≥4.5 cm, as confirmed by magnetic resonance imaging or computed tomography. An interdisciplinary cardiac team evaluated the aortic or valvular complications to determine the most appropriate surgical technique. The surgical procedures employed were the Bentall and de Bono methods, as well as David-5, each chosen based on its level of complexity. The Bentall procedure involves replacing the aortic valve root and ascending aorta with a Dacron tube. Both coronary arteries are anastomosed to the lateral faces of this tube, and one end is attached to a valvular prosthesis. In contrast, the David Type 5 technique preserves the native aortic valve and valve commissures, which are reimplanted within the Dacron tube. For further details, see Table S1 [15].

### Sample and DNA preparation

Peripheral blood samples were obtained through venipuncture into tubes containing sodium ethylenediaminetetraacetic acid (EDTA-Na). White blood cells were isolated from whole blood by lysing erythrocytes using 1X SLR solution. Subsequently, the leukocyte pellet was incubated with 10% SDS and proteinase K (10 mg/mL) at 37 ^∘^C overnight for enzymatic digestion. DNA extraction was performed using the saline expulsion technique. DNA concentration was quantified using a BioPhotometer Plus spectrophotometer at a wavelength of 260/280 nm. The DNA was then purified and concentrated using AMPure XP magnetic beads (Beckman Coulter).

### Gene panel and sequencing experiment

Illumine TruSight Cardio Kit was used to sequence samples, which provides 99% coverage of the regions of 174 genes associated with cardiovascular diseases (TruSight™ Cardio Sequencing Panel), including cardiomyopathies, arrhythmias, aortopathy, and structural heart disease. The TruSight Next Generation Sequencing libraries were prepared according to the manufacturer’s instructions (TruSight™ Cardio Sequencing Panel, Illumina, San Diego, CA, USA). It was necessary to check the size of the DNA fragments prepared with an HS-NGS High Sensitivity 474 kit microchip. Once we observed DNA sizes were approximately 350 bp, we loaded the DNA set at 4 nm to the sequencing cartridge. Pooled libraries were sequenced on NextSeq 550 (Illumina, San Diego, CA, USA) using the paired-ends method with reads of 150 bp.

### Quality controls

Preliminary sequencing data were processed on the Illumina BaseSpace server (https://basespace.illumina.com/home). We calculated the Phred score to assess the accuracy of the base calling. Sequences with a Phred score of less than 30 were excluded from subsequent analyses.

### Annotations of genetic variants

The BAM files, aligned to the GRCh38 reference genome, are hosted on the NCBI server in the S2 repository (https://dataview.ncbi.nlm.nih.gov/object/PRJNA931345?reviewer=og0lqdl3m94ncadi9ruipnemdb). Using NGS, both germline and somatic variants can be characterized for individual patients. After obtaining the aligned and assembled sequences, variant calling was performed using DRAGEN Enrichment software. Variants were annotated using Illumina’s “Variant Interpreter” server (https://variantinterpreter.informatics.illumina.com/home). Only variants that passed the quality control (QC) metrics and had a frequency greater than 0.01 in the TOPmed, 1000 Genomes Project, and NHLBI Exome Sequencing Project were considered. Utilizing the ClinVar database (https://www.ncbi.nlm.nih.gov/clinvar/), which aggregates genomic variation and its relation to human health, we classified the identified genetic variants as pathogenic, likely pathogenic, or variants of unknown significance (VUS). These classifications follow the Standards and Guidelines for the Interpretation of Sequence Variants as recommended by the consensus of the American College of Medical Genetics and Genomics and the Association for Molecular Pathology [16]. Variants were reported according to the nomenclature of the Human Genome Variation Society (HGVS) [17].

### Molecular diagnosis

The genes used for diagnostic criteria included *FBN1* for MFS; for LDS, the diagnostic genes were *TGFBR1-2* and *TGFB1-2*. BHS was diagnosed using the *FBN2* gene, while EDS utilized the *COL5A1* and *COL3A1* genes. WMS was identified using the *TGFB3* gene. Genes associated with arrhythmias, dilated cardiomyopathy, and sudden death were used to diagnose NCTD.

To assess the impact of genetic multipanel testing on the diagnostic certainty of MFS and MFS-like syndromes, we classified patients using four criteria without genetic determination, and a fifth criterion that included not just the *FBN1* gene but also other relevant genes.

### Ethical statement

The study was conducted under the Declaration of Helsinki and approved by the Institutional Review Board (or Ethics Committee) of Instituto Nacional de Cardiologia Ignacio Chavez (registration number INCICh 23-1366). Written consent was obtained from each participant. In the case of minors, verbal approval from the minor and written consent from their legal guardian were required.

### Statistical analysis

The primary analysis was descriptive, employing measures of central tendency to report the results for numerical variables and percentages for dichotomous and nominal variables. Differences were considered statistically significant when the alpha error was less than 0.05. We used STATA Version 16 for the analyses. For mutations with clinical relevance, distance matrices were constructed and represented in heat maps. Hierarchical analyses were conducted using Spearman correlation in RStudio Version 2022.07.1.

## Results

### Characteristics of the study population

This study evaluated 136 patients with the average age ± standard deviation (SD) 24.64 ± 34.75 years, of whom 56 were males, and 80 were females.

The prevalence of diagnosis without multipanel genetic testing was MFS 75, LDS 25, EDS 7, BHS 5, NCTD 21, MASS 2, and WMS 1.

The prevalence of each connective tissue disease classified using the Ghent criteria revised in 2010 (FH, AoD, EL, SS > 7/20 and genetic test) was MFS 64 (47.05%); LDS 16 (11.76%); nonspecific connective tissue disease (NCTD) 32 (23.52%); BHS 13 (9.55%); EDS 10 (7.40%); and WMS 1 (0.72%). We show the demographic characteristics, frequency of comorbidities, and Ghent criteria in Table 1. We also evaluated different cardiovascular conditions using echocardiography and found that the highest prevalence of cardiovascular findings was in MFS and LDS.

**Table 1:** Demographic and anthropometric data of the study population

	**MFS** ***n* ═ 64**	**LDS** ***n* ═ 16**	**NCTD** ***n* ═ 32**	**BHS** ***n* ═ 13**	**EDS** ***n* ═ 10**	**WMS** ***n* ═ 1**
Age (years), median (min–max)	26 (4–59)	22 (4–50)	20 (13–55)	27 (8–39)	23 (10–32)	60
BMI (kg/m^2^), median (min–max)	22 (14–37)	21 (15–24)	20 (13–30)	22 (15–24)	19 (15–23)	28
*Comorbidity, n (%)*						
T2DM	1 (1.5)	0 (0)	0 (0)	0 (0)	0 (0)	0 (0)
SAH	3 (4.6)	1 (6.2)	1 (3.1)	0 (0)	1 (10)	0 (0)
Smoking	2 (3)	0 (0)	1 (3.1)	0 (0)	0 (0)	0 (0)
Obesity	3 (4.6)	0 (0)	0 (0)	0 (0)	0 (0)	0 (0)
Dyslipidemia	3 (4.6)	0 (0)	0 (0)	0 (0)	0 (0)	1 (0)
*Ghent criteria, n (%)*						
Family history	43 (62.2)	5 (31.3)	7 (21.9)	6 (46.2)	1 (10)	0 (0)
Aortic dilatation	51 (79.7)	7 (43.8)	3 (9.4)	2 (15.4)	2 (20)	1 (100)
Ectopia lentis	37 (58)	1 (6.3)	0 (0)	2 (15.4)	1 (10)	0 (0)
Score systemic	62 (96.9)	13 (81.3)	20 (29.4)	12 (92.3)	6 (75)	1 (100)
*Cardiovascular echocardiographic findings, n (%) and median (min–max)*						
MVP	17 (26.6)	2 (12.5)	3 (9.4)	2 (15.4)	4 (40)	0
TVP	4 (6.3)	0 (0)	0 (0)	0 (0)	0 (0)	0
MR	12 (18.8)	3 (18.8)	4 (12.5)	0 (0)	1 (10)	0
AoR	4 (6.3)	1 (6.3)	1 (3.1)	0 (0)	1 (10)	0
TR	13 (20.3)	2 (12.5)	2 (6.3)	1 (7.7)	2 (20)	0
LF	6 (9.4)	1 (6.3)	0 (0)	1 (7.7)	2 (25)	0
SD	1 (1.6)	2 (12.5)	2 (6.3)	1 (7.7)	1 (10)	0
DD	6 (9.4)	3 (18.8)	2 (6.3)	5 (38.5)	4 (40)	1 (100)
LVEF	58 (31–76)	56 (34–69)	62 (48–70)	56 (52–64)	61 (46–66)	60

MFS: Marfan syndrome; LDS: Loeys–Dietz syndrome; NCTD: Nonspecific connective tissue disease, BHS: Beals–Hecht syndrome; EDS: Ehlers–Danlos syndrome; WMS: Weill–Marchesani syndrome; BMI: Body mass index; T2DM: Type 2 diabetes mellitus; SAH: Systemic arterial hypertension; MVP: Mitral valve prolapse; TVP: Tricuspid valve prolapse; MR: Mitral regurgitation, AoR: Aortic regurgitation; TR: Tricuspid regurgitation; LF: Lung failure; LVEF: Left ventricular ejection fraction; SD: Systolic dysfunction; DD: Diastolic dysfunction.

### Genetic mutation

**Figure 1.**
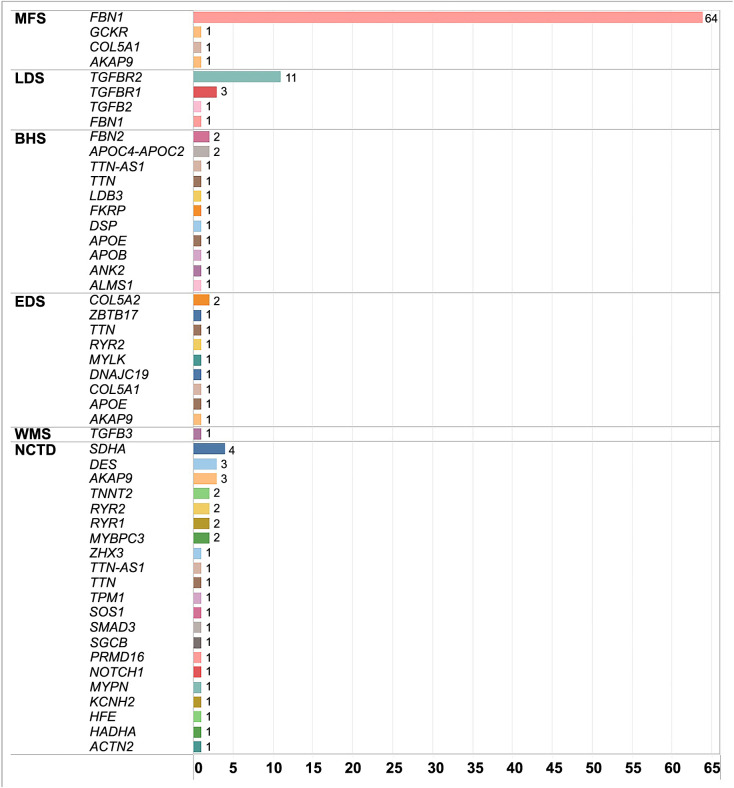
**Mutations reported by NGS in Marfan-like disorders.** Global frequency of reported mutations with clinical value. A total of 64 MFS patients presented mutations in *FBN1*; three patients had, in addition to the *FBN1* gene mutation, mutations in other genes: *AKAP9*, *COL5A1*, and *GCKR.* We found 16 patients with LDS, all with mutations in genes associated with LDS: 11 in *TGFBR2*, three in *TGFBR1*, one in *TGFB2*, and one of them also had a mutation that coexists with *FBN1*. BHS and EDS patients showed mutations in *FBN2* and *COL5A1,* respectively, overlapping with different mutations. The NCTD group showed mutations in genes associated with cardiomyopathies and arrhythmias. NGS: Next-generation sequencing; MFS: Marfan syndrome; LDS: Loeys–Dietz syndrome; BHS: Beals–Hecht syndrome; EDS: Ehlers–Danlos syndrome; WMS: Weill–Marchesani syndrome; NCTD: Nonspecific connective tissue disease; *FBN1:* Fibrillin 1; *TGFBR2:* Transforming growth factor beta receptor 2.

The results obtained from NGS of the 174 target genes evaluated in each patient showed significant variability in mutations across different genes. Therefore, the objective was to identify which patients tested positive for the primary genes involved in and associated with each connective tissue disease. These genes include *FBN1*, *TGFB1-2*, *TGFBR1-2*, *COL5A1*-*COL3A1*, *FBN2*, and *TGFB3*, for the purpose of evaluating and corroborating the clinical diagnosis (Figure 1). Of the patients initially diagnosed with MFS based on meeting more than two criteria, only 64 out of 75 (85%) tested positive for the *FBN1* gene mutation. Three of these patients also had coexisting mutations in other genes: *AKAP9*, *GCKR*, and *COL5A1*. Of the 25 patients clinically classified as having LDS, only 16 out of 25 (64%) had confirmed genetic mutations associated with this syndrome: 11 had mutations in *TGFBR2*, three in *TGFBR1*, one in *TGFB2*, and one had a mutation in both the *FBN1* gene and *TGFBR2*. In the case of BHS, which is initially associated with the *FBN2* gene due to clinical symptoms, five patients had been classified. Of these, only two out of five (40%) were confirmed by *FBN2* gene mutation testing. An additional 11 patients were added to the BHS diagnosis because they had mutations in the *FBN1* gene: eight had initially been classified as having MFS, one had been diagnosed with LDS, and two had NCTD. Besides the *FBN2* gene mutations, some patients also had additional associated genetic mutations, including *ALSM1*, *ANK2*, *APOB*, *APOE*, *DSP*, *LDB3*, *TTN*, and *TTN-AS1*.

Of the seven patients clinically diagnosed with EDS, two out of seven (approximately 30%) were confirmed by genetic testing. An additional eight patients, who initially had other diagnoses, were also confirmed to have EDS through genetic testing. Of these eight patients, one had a mutation only in the *COL5A1* gene, while the other seven had mutations in *COL5A1* along with coexisting mutations in other genes.

Patients diagnosed with NCTD exhibited various types of mutations in genes associated with Brugada syndrome, structural heart disease, long QT syndrome, and familial aortic aneurysm (Figure 1). We analyzed the mutations for diagnostic purposes to see if there were any specific sites or mutagenic patterns. Within the studied cohort, we found that 59% of the mutations were unique, being reported only once, as they were specific to individual patients. Conversely, 41% of the variants were recurrent, appearing in more than one patient. For more details, please refer to the S3 Dataset.

Interestingly, we discovered the *rs79375991* (nucleotide: NM_001024847.3:c.458delA, Exon: 4/8; protein: NM_001024847.2 c.458del [p.Lys153 SerfsTer35]) frameshift indels mutation in the *TGFBR2* gene in 10 of the 16 unrelated patients with LDS. For further information, please refer to Table S2.

Upon conducting the genetic multipanel, we observed that in the group as a whole—comprising both those suspected of having MFS and other connective tissue disorders—the mutation in the *FBN1* gene was present in 64 patients. One patient had mutations in collagen genes, and two had mutations associated with cardiac arrhythmias. In all patients diagnosed with LDS, mutations were found in the *TGFB1*, *TGFB2*, *TGFBR1*, and *TGFBR2* genes. In one instance, coexisting variants were observed in both the *TGFBR2* and *FBN1* genes. It is noteworthy that the *FBN2* gene was mutated in 13 patients; however, in 12 of them, mutations coexisted with other genes associated with MCP, arrhythmias, and FHC. In other words, in 12 out of 13 cases (92%), there was coexistence of mutations. Genes related to MCP and arrhythmias showed high frequencies of 25% and 75%, respectively, in patients classified as having NCTD (Table 2).

**Table 2:** Distribution of patients’ syndromes diagnoses after reclassification according to genetic results

**Dx**	**Aortopathies genes**						**Other genes**			**Total**
	*FBN1 n ═ 65*	*TGFB1-2 TGFBR1-2 n ═ 15*	*TGFB2 n ═ 1*	*TGBR3 n ═ 1*	*COL5A1-2 COL3A1-2 n ═ 11*	*FBN2 n ═ 13*	**MCP genes *n ═ 17***	**Arrhythmias genes *n ═ 30***	**FHC genes *n ═ 5***	**136 (100)**
MFS	64 (100)	–	–	–	1	–	–	2	–	64
LDS	1	15 (93)	1 (7)	–	–	–	–	–	–	16
BHS	–	–	–	–	–	13 (100)	5	2	4	13
EDS	–	–	–	–	10 (100)	–	4	2	1	10
WMS	–	–	–	1 (100)	–	–	–	–	–	1
NCTD	–	–	–	–	–	–	8 (25)	24 (75)	–	32

Numbers in parentheses represent percetages. Dx: Diagnosis; MCP: Myocardiopathies; FHC: Familial hypercholesterolemia; MFS: Marfan syndrome; LDS: Loeys–Dietz syndrome; BHS: Beals–Hecht syndrome; EDS: Ehlers–Danlos syndrome; WMS: Weill–Marchesani syndrome; NCTD: Nonspecific connective tissue disease; *FBN1:* Fibrillin 1.

**Figure 2.**
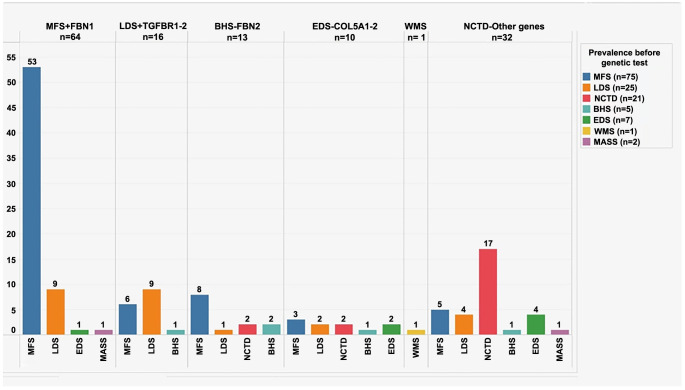
**Diagnosis based on clinical characteristics vs clinical and genetic diagnosis: In the clinical evaluation based on four clinical criteria (AHF, aortic dilatation, ectopia lentis, and systemic score), 75 cases were identified as MFS.** Reclassification of diagnosis was conducted using a multipanel genetic test. For example, out of the 75 initially considered as MFS due to meeting more than two clinical criteria (without the multipanel genetic test), only 64 were classified as MFS after the test. In the MFS + FBN1 column, it is seen that only 53 (71%) were confirmed as MFS, while 22 (29%) were reclassified after the test (highlighted in blue): six as LDS, eight as BHS, three as EDS, and five as NCTD. In this column, in addition to the 53 confirmed as MFS, 11 other patients were reclassified: nine initially diagnosed as LDS, one as EDS, and one as MASS. Similar reclassifications were observed in the other columns corresponding to other diagnoses. The same in the other columns with the other diagnoses. MFS: Marfan syndrome; LDS: Loeys–Dietz syndrome; BHS: Beals–Hecht syndrome; EDS: Ehlers–Danlos syndrome; WMS: Weill–Marchesani syndrome; NCTD: Nonspecific connective tissue disease; MASS: Mitral Valve, Myopia, Skin, and Skeletal disorder; *FBN1:* Fibrillin 1.

In the case of the 64 patients who had a positive test for *FBN1,* our study confirmed that only 53 of those initially clinically classified as MFS were positive for the *FBN1* gene, 53/75 (71%), group (MFS+ FBN1) and another 11 patients initially classified with another diagnosis and who finally had a positive test for the FBN1 mutation were added (Figure 2). Of the 25 patients clinically classified as having LDS, only 16 tested positive for genes associated with LDS (*TGFB1*, *TGFB2*, *TGFBR1*, and *TGFBR2*). In this subset, 9 out of 25 (36%) were confirmed. Furthermore, six patients initially categorized as MFS and one as BHS were reclassified into the group labeled (LDS + *TGFBR1-2*). Regarding the five subjects initially suspected of having BHS, two tested positive for the *FBN2* gene mutation. In the remaining three, genetic testing reclassified them: one as LDS, one as EDS, and another as NCTD. However, 11 additional patients were added to the BHS category because they tested positive for the *FBN2* mutation, confirming their diagnosis of BHS (BHS + *FBN2*). Out of seven patients diagnosed with EDS, only two out of seven (29%) had a positive genetic test for *COL5A1* and *COL3A1*. The genetic multipanel test also identified eight more subjects as having EDS: three initially classified as MFS, two as LDS, two as NCTD, and one as BHS. As a result, we had ten patients in the group labeled (EDS + *COL5A1*/*COL3A1*). For the single patient with WMS, genetic information corroborated the clinical diagnosis. Among the 21 patients initially classified as having NCTD, 17/21 (81%) were confirmed through genetic multipanel testing. Furthermore, 15 cases initially classified as MFS, LDS, EDS, or BHS were reclassified into the NCTD category (Figure 2).

### Genotype–phenotype correlation

**Figure 3.**
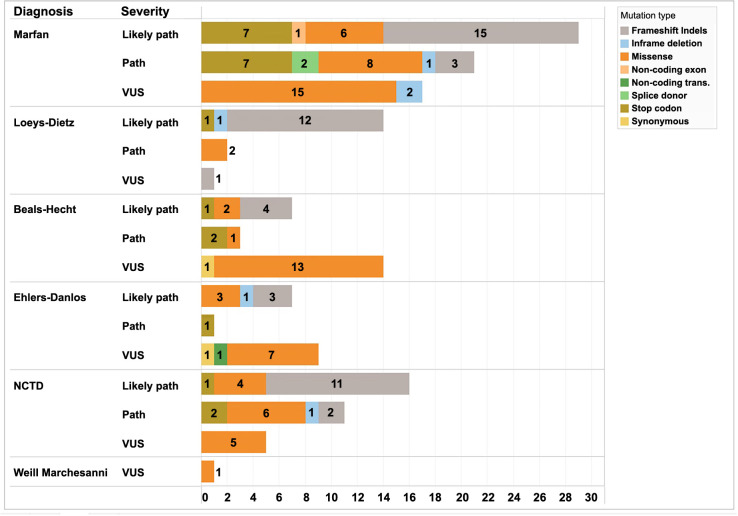
**Type of mutation and reported severity grouped by syndrome studied.** VUS: Variants of unknown significance; NCTD: Nonspecific connective tissue disease; Path: Pathogenic.

The global prevalence of variables of unknown significance (VUS) was 47 (30%), likely pathogenic variants numbered 72 (46%), and pathogenic variants were 39 (24%). The prevalence of the types of mutations was as follows: missense mutations accounted for 73 (46%), frameshift indels for 51 (32%), stop codon mutations for 22 (14%), inframe deletions for six (4%), splice donor mutations for two (1.5%), synonymous mutations for two (1.5%), non-coding transcripts for one (0.5%), and non-coding exons for one (0.5%). Figure 3 shows the prevalence of mutation severity and type according to the diagnosed syndrome. The distribution of the kind of variant in each disease was as follows.

In MFS for VUS, the predominant type of mutation was missense in 15 cases (88%), and two (12%) had inframe deletion. Likely pathogenic variants were frameshift indels in 15 (52%), stop codon in seven (24%), missense in six (21%), and non-coding exon in one case (3%). In subjects with pathogenic variants, there was a higher prevalence of missense in eight (38%), stop codon in seven (33%), frameshift indels in three (14%), splice donor in two (10%), and inframe deletion in one case (5%).

In LDS, VUS variants had one case with missense mutations. Likely pathogenic variants were frameshift indels in 12 (86%), stop codon in one (7%), and inframe deletion in one case (7%). Pathogenic variants were two with (100%) missense.

In BHS, VUS variants were missense in 13 (93%) and synonymous in one case (7%). Likely pathogenic presents frameshift indels in four (57%), missense in two (29%), and stop codon in one case (14%). Pathogenic variants show stop codon in two (67%) and missense in one case (33%).

EDS in VUS had missense in seven, synonymous in one, and non-coding transcript in one. Likely pathogenic shows missense in three (43%), frameshift indels in three (43%), and inframe deletion in one case (14%). The pathogenic variant was stop codon in one case (100%).

In patients with NCTD, according to the severity of variants in VUS, five cases had missense, likely pathogenic were: frameshift indels in 11, missense in four, and stop codon in one; pathogenic variant had missense in six, stop codon in two, frameshift indels in two, and inframe deletion in one case.

**Figure 4.**
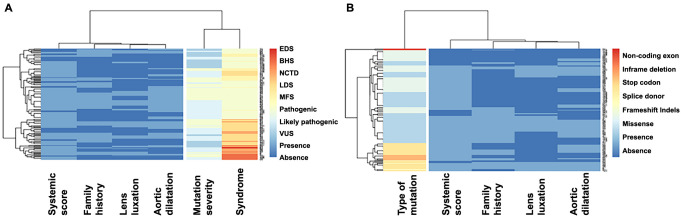
**Distance matrix represented in a heat map**. (A) Mutation severity and the four Ghent criteria; (B) Type of mutation and the four Ghent criteria. Clinical criteria are presented as presences (light blue) and absences (dark blue). Clinical criteria are depicted with color differentiation—presence in light blue and absence in dark blue. Each row represents an individual patient, allowing for a patient–centric view of the data. As you move horizontally from left to right, the color intensity (ranging from intense to low blue) signifies the presence or absence of a Ghent clinical criterion. Further to the right, a column with a distinct color represents the severity of the variant, followed by another column indicating the type of syndrome. For statistical interpretation, two specific columns are of focus—one identifying the type of syndrome and the other visually representing the severity of the variant. By examining these columns, one can discern, from right to left, the proportion of patients with MFS exhibiting a particular variant type, which, in turn, correlates with the presence of certain clinical criteria. MFS: Marfan syndrome; LDS: Loeys–Dietz syndrome; BHS: Beals–Hecht syndrome; EDS: Ehlers–Danlos syndrome; NCTD: Nonspecific connective tissue disease; VUS: Variants of unknown significance**.**

To investigate the association between the severity of mutations and the types of mutations in relation to the Ghent clinical criteria, we constructed a distance matrix. In the statistical analysis, we utilized Spearman correlations, which are visually represented in a heat map (Figure 4).

Regarding the severity of mutations, our analysis revealed the following correlations.

In MFS, there was a low correlation between pathogenic severity and AoD, with *r*^2^ ═ 0.27 (*P* ═ 0.02). In patients with LDS, we found a strong correlation between VUS severity and SS (*r*^2^ ═ 0.79, *P* ═ 0.001), as well as between pathogenic severity and the presence of the *FBN1* gene (*r*^2^ ═ 0.68, *P* ═ 0.004). In patients with BHS, correlations were identified between likely pathogenic severity and both EL and AoD (*r*^2^ ═ 0.67, *P* ═ 0.01) for both conditions (Table 3).

**Table 3:** Correlation between the severity of variants and the Ghent criteria

	**Family history**		**Ectopia lentis**		**Aortic dilation**		**Systemic score**		*FBN1*	
	*r* ^**2**^	*P*	*r* ^**2**^	*P*	*r* ^**2**^	*P*	*r* ^**2**^	*P*	*r* ^**2**^	*P*
*MFS*										
VUS	−0.12	0.33	0.13	0.33	−0.18	0.15	0.15	0.21	−0.15	0.21
Likely pathogenic	0.10	0.41	−0.08	0.58	−0.133	0.51	−0.03	0.78	−0.03	0.78
Pathogenic	−0.009	0.78	−0.01	0.94	0.27	0.02	0.15	0.22	0.15	0.22
*LDS*										
VUS	0.13	0.62	−0.09	0.71	0.42	0.09	0.79	0.0001	−0.09	0.71
Likely pathogenic	−0.21	0.44	0.14	0.58	−0.07	0.58	0.46	0.07	−0.44	0.07
Pathogenic	0.13	0.62	−0.09	0.71	−0.33	0.20	0.18	0.50	0.68	0.004
*EDS*										
VUS	−0.27	0.44	−0.27	0.44	−0.41	0.24	0.25	0.48	0.00	1
Likely pathogenic	0.33	0.44	0.33	0.34	0.50	0.14	−0.40	0.24	0.00	1
Pathogenic	−0.11	0.78	−0.11	0.76	−0.16	0.76	0.27	0.44	0.00	1
*BHS*										
VUS	−0.07	0.81	−0.46	0.11	−0.46	0.11	0.31	0.11	0.00	1
Likely pathogenic	−0.26	0.37	0.67	0.01	0.67	0.01	0.08	0.78	0.00	1
Pathogenic	0.22	0.47	0.10	0.41	0.10	0.74	−0.36	0.22	0.00	1
*NCTD*										
VUS	0.26	0.13	0.00	1	0.08	0.62	−0.01	0.99	0.00	1
Likely pathogenic	−0.14	0.42	0.00	1	0.06	0.71	−0.23	0.18	0.00	1
Pathogenic	−0.09	0.59	0.00	1	−0.17	0.35	0.29	0.10	0.00	1

*r*^2^ ═ Spearman correlation; *P* ═ alfa error <0.05; MFS: Marfan syndrome; LDS: Loeys–Dietz syndrome; BHS: Beals–Hecht syndrome; EDS: Ehlers–Danlos syndrome; NCTD: Nonspecific connective tissue disease, VUS: Variants of unknown significance; *FBN1:* Fibrillin 1.

We observed the following correlations with regard to the type of mutation.

In MFS, missense mutations were correlated with EL (*r*^2^ ═ 0.27, *P* ═ 0.03), while frameshift indels were associated with a FH (*r*^2^ ═ −0.29, *P* ═ 0.01). In LDS, the type of mutation was correlated with both FH (*r*^2^ ═ 0.53, *P* ═ 0.04) and AoD (*r*^2^ ═ 0.49, *P* ═ 0.05). In patients with BHS, missense mutations were associated with the presence of a SS (*r*^2^ ═ 0.67, *P* ═ 0.01). Stop codon mutations correlated with the presence of ectopia lenticularis (*r*^2^ ═ 0.67, *P* ═ 0.01) and AoD (*r*^2^ ═ 0.67, *P* ═ 0.01). In patients with undifferentiated connective tissue disease, frameshift indels and inframe deletions were associated with the presence of SSs (*r*^2^ ═ 0.43, *P* ═ 0.01) and (*r*^2^ ═ 0.48, *P* ═ 0.005), respectively. For further details, see Table 4.

**Table 4:** Correlation between the type of variants and the Ghent criteria

	**Family history**		**Ectopia lentis**		**Aortic dilation**		**Systemic score**		*FBN1*	
	*r* ^**2**^	*P*	*r* ^**2**^	*P*	*r* ^**2**^	*P*	*r* ^**2**^	*P*	*r* ^**2**^	*P*
*MFS*										
Missense	0.16	0.18	0.27	0.03	−0.11	0.35	0.00	–	0.00	1
Stop codon	−0.007	0.95	−0.22	0.08	0.14	0.25	−0.08	0.49	0.11	0.37
Frameshift Indels	−0.29	0.01	0.02	0.83	−0.01	0.88	0.07	0.56	−0.18	0.14
Inframe deletion	0.08	0.48	−0.15	0.23	0.06	0.61	0.02	0.85	0.02	0.85
Splice donor	−0.06	0.60	0.15	0.23	0.09	0.47	0.03	0.80	0.03	0.80
Non-coding exon	–	–	–	–	–	–	–	–	–	–
Synonymous	–	–	–	–	–	–	–	–	–	–
*LDS*										
Missense	0.53	0.04	−0.17	0.51	0.49	0.05	0.36	0.16	−0.17	0.50
Stop codon	0.009	1	0.00	–	–	–	–	–	–	–
Frameshift Indels	−0.40	0.14	0.20	0.45	−0.35	0.17	0.28	0.27	−0.33	0.20
Inframe deletion	0.00	–	–	–	–	–	–	–	–	–
Splice donor	−0.18	0.50	−0.06	0.80	−0.22	0.39	0.12	0.64	–	–
Non-coding exon	–	–	–	–	–	–	–	–	–	–
Synonymous	–	–	–	–	–	–	–	–	–	–
*EDS*										
Missense	−0.33	0.34	−0.33	0.34	−0.50	0.14	0.40	0.24	–	–
Stop codon	0.00	1	0.33	0.34	–	–	–	–	–	–
Frameshift Indels	0.33	0.34	0.33	0.34	0.50	0.14	−0.40	0.24	–	–
Inframe deletion	0.00	–	–	–	–	–	–	–	–	–
Splice donor	0.00	–	–	–	–	–	–	–	–	–
Non-coding exon	–	–	–	–	–	–	–	–		
Synonymous	–	–	–	–	–	–	–	–		
*BHS*										
Missense	−0.03	0.91	−0.40	0.16	−0.40	0.16	0.67	0.01	–	–
Stop codon	−0.26	0.37	0.67	0.01	0.67	0.01	0.08	0.79	–	–
Frameshift Indels	0.00	–	–	–	–	–	–	–	–	–
Inframe deletion	0.00	–	–	–	–	–	–	–	–	–
Splice donor	0.00	–	–	–	–	–	–	–	–	–
Non-coding exon	0.31	0.30	−0.12	0.68	−0.12	0.68	0.00	1	–	–
Synonymous	–	–	–	–	–	–	–	–	–	–
*NCTD*										
Missense	0.08	0.64	0.00	–	0.08	0.62	0.14	0.43	–	–
Stop codon	−0.13	0.45	0.00	–	−0.08	0.62	0.03	0.86	–	–
Frameshift Indels	0.04	0.81	0.00	–	0.08	0.63	−0.43	0.01	–	–
Inframe deletion	−0.02	0.91	–	–	−0.13	0.45	0.48	0.005	–	–
Splice donor	−0.02	0.91	–	–	–	–	–	–	–	–
Non-coding exon	–	–	–	–	–	–	–	–	–	–
Synonymous	−0.09	0.60	0.00	1	−0.05	0.65	−0.15	0.38		

*r*^2^ ═ Spearman correlation; *P* ═ alfa error <0.05; MFS: Marfan syndrome; LDS: Loeys–Dietz syndrome; BHS: Beals–Hecht syndrome; EDS: Ehlers–Danlos syndrome; NCTD: Nonspecific connective tissue disease.

To investigate the relationship between the severity of heart conditions requiring surgical intervention and specific mutations, we selected patients who had undergone surgery at the National Institute of Cardiology. Data were extracted from the institute’s clinical records. Table 5 presents the results: out of 136 participants, 17 underwent surgery to modify or preserve the aortic valve in the past ten years. Among these, 14 were diagnosed with MFS; five had pathogenic variants and nine had likely pathogenic variants.

**Table 5:** List of mutations and their severity in all the operated for aortic aneurism patients

**Case**	**Type of surgery**	**Age at surgery**	**Dx**	**Gene**	**Severity of mutation**	**Type of mutation**	**Location**
393	David	27	MFS	*FBN1*	Path	Stop codon	c.4621C>T (p.Arg1541Ter); Exon: 38/66
365	David	22	MFS	*FBN1*	Path	Stop codon	c.4621C>T (p.Arg1541Ter); Exon: 38/66
204	Bentall and de Bono	49	MFS	*FBN1*	Path	Frameshift Indels	c.1957_1958del (p.Val653Ter); Exon: 16/66
203	Bentall and de Bono	40	MFS	*FBN1*	Path	Frameshift Indels	c.7039_7040del (p.Met2347 ValfsTer19); Exon: 58/66
239	David	35	MFS	*FBN1*	Path	Stop codon	c.326C>T(p.Arg2776Ter); Exon: 66/66
265	Aortic valve replacement	32	MFS	*FBN1*	Likely path	Missense	c.6331T>C (p.Cys2111Arg); Exon: 52/66
209	David	4	MFS	*FBN1*	Likely path	Frameshift Indels	c.5846dup (p.Asn1949LysfsTer12); Exon: 48/66
229	Bentall and de Bono	24	MFS	*FBN1*	Likely path	Stop codon	c.3697C>T (p. Gln1233Ter); Exon: 30/66
366	Florida sleeve	27	MFS	*FBN1*	Path	Missense	c.5503 T>C (p.Cys1835Arg); Exon: 45/66
276	Bentall and de Bono	38	MFS	*FBN1*	Likely path	Frameshift Indels	c.8516del (p.Lys2839ArgfsTer7); Exon: 66/66
242	Dacron	20	MFS	*FBN1*	Likely path	Missense	c.7134C>G (p.Cys2378Trp); Exon: 58/66
294	Bentall and de Bono	17	MFS	*FBN1*	Likely path	Stop codon	c.177 T>A(p.Cys59Ter); Exon: 3/66
329	Bentall and de Bono	18	MFS	*FBN1*	VUS	Missense	c.5728G>T (p.Gly1910Cys); Exon: 47/66
295	Dacron	31	MFS	*FBN1*	Likely path	Missense	c.1427G>A (p.Cys476Tyr); Exon: 12/66
219	Florida sleeve	29	LDS	*TGFB2*	Likely path	Frameshift Indels	c.458del (p.Lys153SerfsTer35); Exon: 4/8
300	Florida sleeve	48	LDS	*TGFB1*	Likely path	Missense	c.769A>G (p.Met257Val); Exon: 4/9
171	David	20	EDS	*COL5A2*	Likely path	Frameshift Indels	c.2355del (p.Gly786ValfsTer3); Exon: 35/54

Dx: Diagnosis; Path: Pathogenic; MFS: Marfan syndrome; LDS: Loeys–Dietz syndrome; EDS: Ehlers–Danlos syndrome; *FBN1:* Fibrillin 1; VUS: Variants of unknown significance.

Upon examining the locations of these mutations, we found only one recurring mutation, a stop codon, in two patients: *rs794728228* (NM_000138.4:c.4621C>T [p.Arg1541Ter], Exon: 38/66). This observation is particularly noteworthy as the patients are siblings who both required surgical intervention.

## Discussion

The revised version of the Ghent nosology, introduced in 2010, provides key criteria for diagnosing MFS with excellent specificity. However, even with these criteria focusing specifically on genetic testing of the *FBN1* gene, their utility is limited when it comes to differentiating MFS from other disorders, such as WMS, EDS, BHS, and LDS. To enhance diagnostic precision, it may be beneficial to incorporate a genetic multipanel study that examines genes other than just FBN1 [10].

While genetic testing for the *FBN1* gene, as suggested by the Ghent criteria, improves the likelihood of diagnosing MFS, it is insufficient for ruling out other Marfan-like connective tissue disorders. For this reason, the same revised Ghent nosology recommends supplementing genetic tests with analyses of *TGFBR1*, *TGFBR2*, *COL3A1*, and collagen biochemistry, especially when symptoms of MFS overlap with those of other connective tissue disorders. Our findings indicate that a genetic multipanel study offers comprehensive insights for patients with connective tissue diseases, given that significant clinical heterogeneity often complicates classification.

Classification of patients with MFS is generally based on the Ghent criteria. However, some patients who meet two or more clinical criteria may test negative for *FBN1* mutations but possess mutations in other genes. Conversely, another subset of patients meets the clinical criteria and tests positive for *FBN1* mutations, but also coexist with mutations in other genes. The implications of these findings could enhance our understanding of the phenotypic and genotypic complexities in these patients. Although our study yields intriguing data, the results are not yet fully explained.

Patients with connective tissue diseases often present with syndromic conditions, making both phenotype and genotype highly variable and challenging for clinical judgment. Therefore, it is advisable to conduct a comprehensive genetic multipanel study. Such a study could offer insights beyond the protein encoded by *FBN1*, as other proteins interacting within the microfibril network of the extracellular matrix may also contribute to the pathology [18].

LDS is categorized into six types based on clinical characteristics and their association with specific mutations in the *TGFBR2*, *TGFBR1*, and *TGFB2 SMADS* genes. Given the clinical and genetic heterogeneity within this patient group, a multipanel genetic study could provide a more accurate diagnosis for each individual. Such an approach may also identify cases of genetic coexistence, as observed in clinical settings.

Our findings demonstrate that the use of a genetic multipanel significantly impacts the classification of patients. We emphasize the importance of conducting comprehensive patient evaluations using this method. In most cases, clinical observations alone are sufficient for the preliminary diagnosis of MFS [19]. However, genetic testing enables us to confirm or reclassify these diagnoses [20]. There is a notable clinical overlap among various connective tissue disorders, and timely identification of each allows for improved patient survival. In the case of patients with LDS, short-term life prognosis is generally poor due to rapidly progressive and lethal cardiovascular complications. An accurate diagnosis through genetic testing could thus lead to more stringent monitoring and timely interventional management [21, 22]. The multipanel approach may offer substantial support to physicians during clinical trials, treatment, and patient follow-up. Our results indicate that LDS has a high prevalence rate of mutations in the *TGFB1* and *FBN1* genes. Intriguingly, 63% (10/16) of unrelated patients diagnosed through genetic testing displayed the same rs79375991 mutation, located in exon 4 (NM_001024847.2c.458del [p.Lys153SerfsTer35] Exon: 4/8).

This variant results in an amino acid alteration, replacing a lysine (K) with a serine (S) at position 128 and creating a premature stop signal in the new reading frame (K128Sfs*35). In-silico analysis predicts that this substitution will result in a non-functional TGFBR2 protein. While no research specifically focuses on this mutation, some studies have reported it in familial cases of bicuspid aortic valve [23]. Additionally, the most common congenital heart defects reported in children with LDS types one and two include bicuspid aortic valve, Tetralogy of Fallot, patent ductus arteriosus, and atrial septal defect [24, 25].

Upon reviewing the clinical records of patients with LDS spectrum disorders who have the rs79375991 mutation, we found a high prevalence of bicuspid aortic valve (6/9 patients). This mutation is located in exon 4, in the polyA section affected by the reading frame. Due to the location of this variant within the *TGFBR2* locus, it results in decreased TGFBR2 protein levels and reduced TGFβ signaling. This could potentially provide a mechanism for the development of LDS. Exhaustive experiments are needed to corroborate the relationship between this variant and the development of LDS in conjunction with a bicuspid aortic valve [26]. It is crucial to note that the other six patients presented mutations at different sites within the *TGFB1* and *TGFBR1-2* genes. This means that 37% of the mutations in our LDS cohort exhibit significant variability, which could reflect phenotypic variability. Although we employ genetic tests to identify NGS mutations and aim to make a molecular diagnosis, the role of genetic testing in establishing diagnoses needs further refinement [27].

In the case of patients with MFS, we found a great variability of sites that present mutations, so we focused our attention on MFS with more severe stages of aortic aneurysms. Upon reviewing the clinical data, we found that 14 out of the 17 patients who underwent surgical correction of the aortic valve had a diagnosis of MFS. In terms of mutations in the *FBN1* gene, there was substantial variability. However, an exception was noted in two siblings who shared the same mutation, rs794728228 (NM_000138.4c.4621C>T [p. Arg1541Ter] Exon: 38/66), a result consistent with Mendelian inheritance. Interestingly, despite sharing the same genotype, their phenotypes differed: one required David’s procedure, while the other needed an aortic valve replacement. This suggests that even among family members with the same *FBN1* mutation, the manifestation of aortic damage can vary. This highlights the need for further investigation into additional factors that may contribute to or coexist with the damage mechanism [28, 29]. It indicates that a single pathogenic variant in a single gene may not solely determine phenotype severity. Therefore, we must consider the possibility that the lack of a genotype–phenotype association could be due to the presence of mutations in multiple genes—*FBN1*, *TGBR1*, *COL5*, *COL5A1*, and *FBN2*—in the same patient. To draw a more comprehensive correlation, consideration should also be given to genes associated with musculoskeletal and ocular malformations [30].

On the other hand, in various cohorts, mutations in exons 24–32 have been identified as critical regions for the neonatal form of MFS, characterized by severe mitral or tricuspid valve regurgitation and pulmonary emphysema [31]. In our study of the Mexican population, we discovered mutations in different exons of the *FBN1* gene in patients with severe aortic dilation requiring surgical intervention. Although we did not identify a specific site associated with more advanced stages of MFS, our review of the types of mutations in patients who underwent surgery revealed a high prevalence of stop codon and frameshift indels mutations—accounting for 65% of the cases. These findings align with various reports suggesting that frameshift insertions or deletions in the *FBN1* gene in Marfan patients are linked to more severe clinical manifestations of the disease [32, 33]. Furthermore, mutations leading to a premature stop codon are also associated with more severe forms of the disease [34]. The relationship of these mutations to nonsense-mediated mRNA decay warrants further investigation to determine if they are linked to more severe aortic phenotypes compared to those with missense variants [35]. However, while mutations may occur at different sites within a gene, the pathogenicity of each mutation should be carefully considered in clinical decision making, especially when determining the need for surgical intervention for aneurysms based on various aortic segments’ diameters. These findings suggest that genetic factors may play a significant role and should be considered alongside other variables, such as excess aortic length, FH, aortic stress, patient height, arterial tortuosity, root location, inflammation, bicuspid aortic valve, and existing genetic mutations [36]. The heat maps indicate that in our entire population, we did not find associations between genetic results and a higher number of Ghent criteria among the patients. This complicates the genotype–phenotype association in MFS. Known clinical heterogeneity has been a hurdle for this type of study. It is likely that epigenetic and environmental factors, along with genetic modifiers, play a significant role in the clinical variability of MFS and related syndromes [37]. The majority of mutations exhibit single nucleotide polymorphisms (SNPs), which could be one reason we did not find an association in the context of two FBN1 alleles [38]. Evidence suggests that there is a higher likelihood of half-normal production of the normal protein (haploinsufficiency) rather than the production of a mutant protein. This could be crucial for the clinical expression of the disorder [39]. To test this hypothesis, it would be necessary to quantify mRNA expression in patients with FBN1 mutations and correlate this with protein expression data.

In the case of patients classified as having NCTD, we did not identify genes directly associated with any specific connective tissue disease. Nevertheless, these patients exhibit clinical characteristics resembling Marfan-like syndromes. Data obtained through NGS indicate that the gene mutations in this group are associated with arrhythmias and cardiomyopathy, leading to sudden death. For this reason, we believe it is essential to focus on these patients, who are classified as having undifferentiated connective tissue diseases. This is a population with the highest frequency of gene mutations related to sudden death and arrhythmogenic cardiomyopathy of the left ventricle. Therefore, careful patient selection by an interdisciplinary team specializing in these rare conditions will be necessary for conducting pertinent research. This will help determine the significance of these genetic mutations in causing cardiovascular damage. The limitations of this study include the rarity of the diseases under investigation, which restricts our ability to assemble large patient cohorts and hampers evaluation with larger samples of patients exhibiting conditions similar to MFS. Additionally, it was not possible in this study to incorporate other genes associated with aortic and cardiovascular damage, as well as genes related to musculoskeletal, ocular, and metabolic injuries. These additional genes could have provided further insight into the phenotype–genotype association.

This series represents one of the most extensive studies involving patients from our country, given that it was conducted at an Institute of Cardiovascular Care, which also serves as a reference center for treating these rare conditions and is dedicated to research. This study has enabled us to present findings on Mexican patients with connective tissue disorders, a subject on which there have been no prior reports. The advancements made in this project contribute valuable data to translational research.

## Conclusion

The genetic multipanel study plays a significant role in the initial evaluation of patients with MFS and other Marfan-like conditions. Our findings indicate that genetic testing provides greater diagnostic certainty than relying solely on clinical aspects. Additionally, identifying the severity and type of genetic variant can inform clinical decision making for patients who may require interventional or surgical treatment.

The correlation between phenotype and genotype in these disorders, as assessed by the genetic panel, is complex due to significant heterogeneity in both phenotype–genotype associations and the diversity of aortic and cardiovascular damage. A future perspective could involve expanding the multipanel to include genes related to musculoskeletal, ocular, and metabolic impairments in these syndromes.

## Supplemental data

Supplementary data are available at the following link: https://www.bjbms.org/ojs/index.php/bjbms/article/view/9578/2934
